# Prevalence of *Capillaria plica* in Danish wild carnivores

**DOI:** 10.1016/j.ijppaw.2018.09.006

**Published:** 2018-09-22

**Authors:** Heidi H. Petersen, Stine T. Nielsen, Gitte Larsen, Elisabeth Holm, Mariann Chriél

**Affiliations:** Section for Diagnostics and Scientific Advice, National Veterinary Institute, Technical University of Denmark, Kemitorvet, 2800, Kgs. Lyngby, Denmark

**Keywords:** *Capillaria plica*, Bladderworm, Wild carnivores, Reservoir hosts

## Abstract

*Capillaria plica* is a parasitic nematode belonging to the family Capillariidae. The adult parasites reside in the urinary tract of wild and domestic canines. The infection is most often asymptomatic, but can cause a wide range of symptoms including urinary bladder inflammation, pollacisuria, dysuria and hematuria. Canines acquire the infection by ingesting the intermediate host, the earthworm (Lumbricidae). Epidemiological studies on *C. plica* infection in wildlife are few and only one previous Danish study examined the prevalence in red foxes, while studies on prevalence in other animals are limited. We examined the urine sediment or urinary bladder from 375 Raccoon dogs (*Nyctereutes procyonoides*), 247 red foxes (*Vulpes vulpes*), 20 beech martens (*Martes foina*), 16 wild mink (*Neovison vison*), 14 otters (*Lutra lutra*), nine European polecats (*Mustela putorius*), three European badgers (*Meles meles*) and one golden jackal (*Canis aureus*) received as a part of Danish wildlife surveillance. *Capillaria plica* was detected in 73.7% of red foxes, 20.0% of beech martens, 0.5% of raccoon dogs, and in the Golden jackal. Red foxes originating from all 5 regions of Denmark were infected, although with a significantly higher prevalence in the three regions in Jutland compared to Region Zealand.

## Highlights

• *Capillaria plica* prevalence was high in Danish red foxes.
• *Capillaria plica* prevalence was low or non-existent in other wild carnivores.
• This study documents *C. plica* infection in beech martens for the first time.

## Graphical abstract

**Figure:**
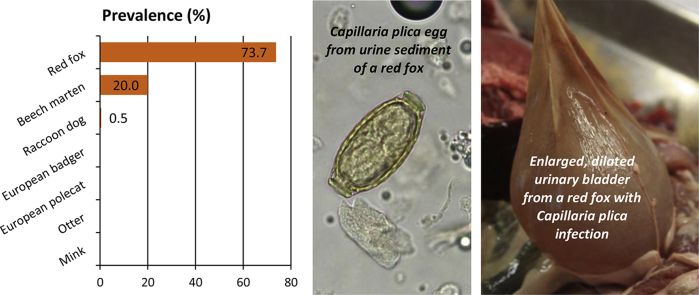

## 1 Introduction

**Fig. 1:**
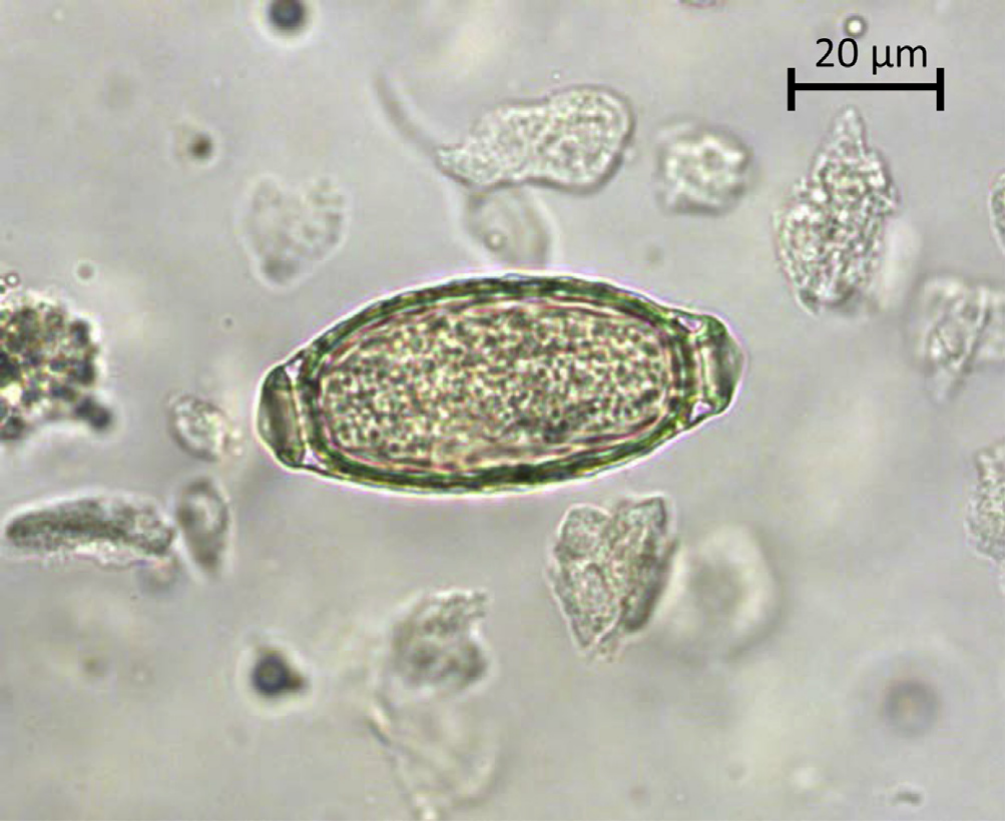
A typical barrel-shaped *Capillaria plica* egg in urine sediment from a red fox. The egg show a slightly pitted shell and two opercules with polar plugs. (For interpretation of the references to colour in this figure legend, the reader is referred to the Web version of this article.)

The bladderworm *Capillaria plica* (syn. *Pearsonoma plica*) is a threadlike nematode belonging to Capillariidae family (Butterworth and Beverley-Burton, 1980). The life-cycle is indirect, involving wild and domestic canines as final host, and earthworms (Lumbricidae) as intermediate host. The final host become infected when ingesting earthworms containing first-stage larvae (L1). The L1 develop to third-stage larvae (L3) in the small intestine, migrate to the urinary bladder and moult to adult worms within two months. Adults are 13–60 mm long and embedded in the bladder mucosa. Occasionally, adults reside in urethra and renal pelvis. The female worm lays 55–67 × 26–29 μm barrel-shaped eggs with buttons on both poles (Fig. 1). The eggs are spread to the environment with urine.

*Capillaria plica* infection is generally asymptomatic, but severe urinary bladder inflammation, glomerular amyloidosis, oedema, hyperplasia of the mucosal membranes leading to pollacisuria, dysuria and hematuria are documented in dogs (Callegari et al., 2010; Lamrna and Main, 1964; Senior et al., 1980).

In Europe, *C. plica* infection is common in red foxes with reported prevalences of 78% in Germany (Bork-Mimm and Rinder, 2011), 53% in Norway (Davidson et al., 2006) and 52% in Hungary (Sréter et al., 2003). In Denmark, the prevalence has previously been documented to 80.5% in 748 red foxes collected over a five-year period in 1997–2002 (Saeed et al., 2006). Studies on *C. plica* infections in other carnivores are scarce, and no other Danish wild carnivores have previously been examined for *C. plica*.

The purpose of this study was to carry out a nation-wide cross-sectional study of *C. plica* prevalence in Danish wild carnivores, to determine their role as reservoir host for infection in Danish dogs.

## 2 Material and methods

The urinary bladder was obtained from 375 raccoon dogs (*Nyctereutes procyonoides*), 247 red foxes (*Vulpes vulpes*), 20 beech martens (*Martes foina*), 16 mink (*Neovison vison*), 14 otters (*Lutra lutra*), nine European polecats (*Mustela putorius*), three European badgers (*Meles meles*) and one golden jackal (*Canis aureus*).

The carcasses of the animals were submitted to the National Veterinary Institute, Technical University of Denmark (DTU-VET) as a part of general wildlife surveillance in 2017. The animals were either found dead, euthanized for animal welfare reasons, or hunted. The dead carnivores were transported in sealed plastic bags at −20 °C to DTU-VET and left at −80 °C for ≥2 days to inactivate potential infective parasites before necropsy. The region was listed along with information on date of death if recorded. Urinary bladders were recovered and placed in liquid-tight plastic boxes. The bladders were carefully opened and 10 ml of urine collected. If urine was absent, the bladder was cut open (n = 586), washed with 10 ml milliQ water and the water collected in 15 ml plastic tubes. All samples were centrifuged (178 × g) for 10 min, the supernatant discarded to 1.5 ml, flotation fluid added to 3 ml, and samples screened for *C. plica* eggs (Fig. 1). The egg quantity per ml was recorded.

The prevalence of *C. plica* infection per animal species, and per region for red foxes was calculated together with the 95% confidence intervals. Difference in *C. plica* prevalence for red foxes between the five regions was determined by a binary logistic regression model with *C. plica* infection as the dependent variable and region of origin as the independent variable. A p-value of 0.05 was considered significant.

## 3 Results

*Capillaria plica* eggs were isolated from red foxes, raccoon dogs, beech martens and the golden jackal. Mink, otters, European polecats and European badgers were negative for *C. plica*. In total, 182 red foxes harboured *C. plica*, corresponding to a prevalence of 73.7%. Of the 375 examined raccoon dogs, only two were positive, corresponding to a prevalence of 0.5%. Of the 20 beech martens examined, four were positive (20%).

Table 1 shows the egg load (eggs per ml) in the urine or urinary bladder of positive animals (n = 189). More than half of the positive red foxes (n = 94, 52.8%) had an egg load above 30 eggs per ml.

**Table 1:** *Capillaria plica* egg load in urine sediment or sediment from washing of the bladder.

Species	No examined	No positive animals (%)	95% confidence interval	1-10 eggs per ml (%)	11-20 eggs per ml (%)	21-30 eggs per ml (%)	>30 eggs per ml (%)
Red foxes (*Vulpes vulpes*)	247	182 (73.7)	68.2–79.2	41 (23.0)	31 (17.4)	12 (6.7)	94 (52.8)
Raccoon dogs (*Nyctereutes procyonoides)*	375	2 (0.5)	0.2–1.3	1 (50.0)	1 (50.0)	–	–
Golden jackal (*Canis aureus*)	1	1 (100.0)	–	–	–	–	1 (100.0)
Beech martens (*Martes foina*)	20	4 (20.0)	0.8–39.2	–	4 (100.0)	–	–

**Fig. 2:**
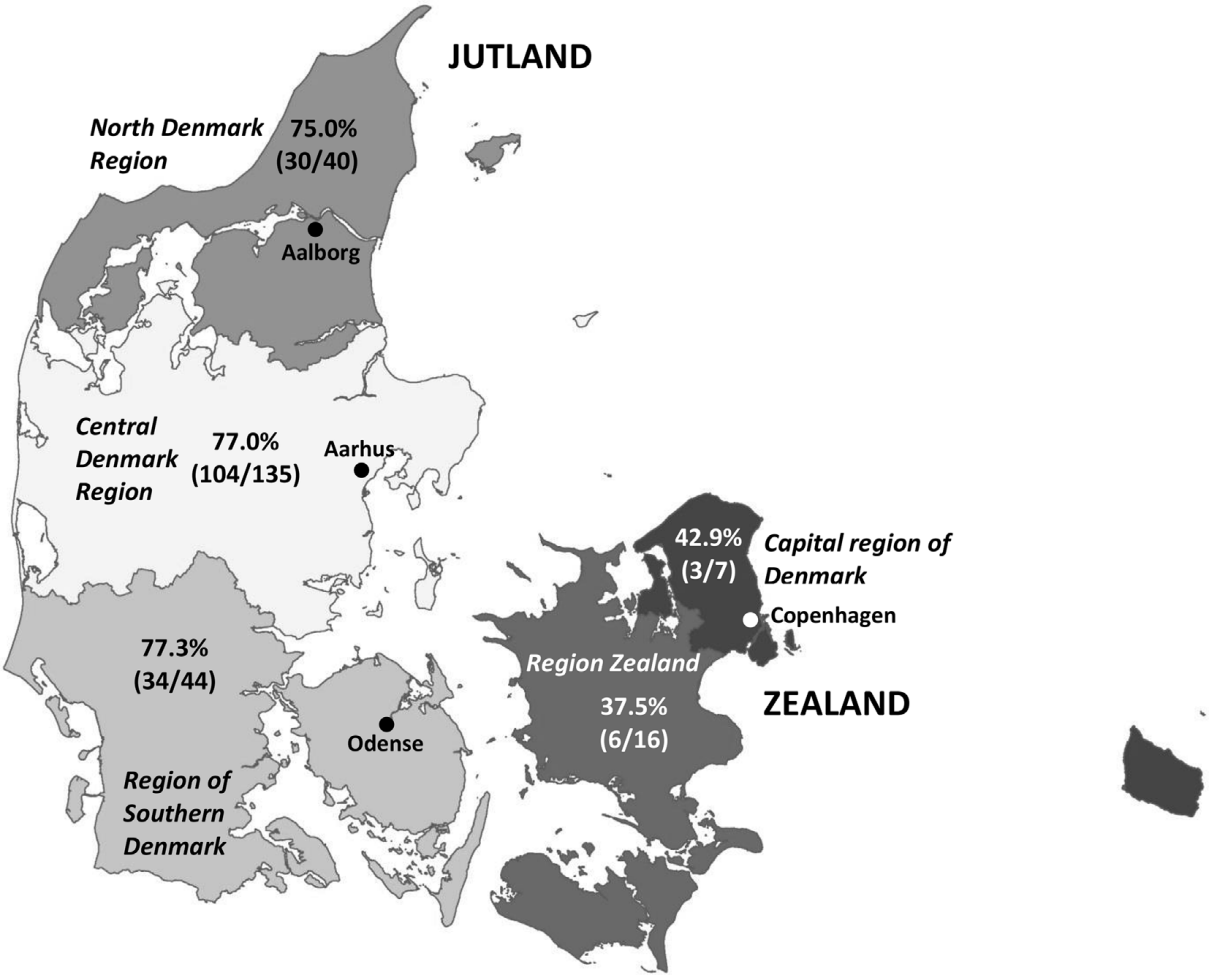
The prevalence of *Capillaria plica* infections in red foxes per region of Denmark (positive red foxes/total number of red foxes examined). The origin of five red foxes was unknown.

Fig. 2 show the prevalence of *C. plica* positive red foxes in the five Danish regions. In the three regions of Jutland (north Denmark region, central Denmark region and region of southern Denmark), *C. plica* prevalence was above 75%, while the prevalence in red foxes from the two regions on Zealand (region Zealand and capital region of Denmark) it was below 50% (Fig. 2). However, only in region Zealand, the *C. plica* prevalence was significant lower (p < 0.05) than the prevalence in the three regions in Jutland.

## 4 Discussion

In this cross-sectional study of *C. plica* infection in Danish wild carnivores, we identified red foxes as the most likely reservoir host for infection in dogs, as *C. plica* infection was common in red foxes (73.7%). In contrast, *C. plica* infection was rare in raccoon dogs, and absent in wild mink, otters, polecats and badgers. Lastly, we report *C. plica* infection in beech martens for the first time.

The high prevalence in red foxes in our study corresponds with the previous finding in Denmark (80.5%), although data were collected 16–21 years ago (Saeed et al., 2006). This demonstrates that *C. plica* continues to be highly prevalent in Danish red foxes and that red foxes most likely are the main definitive host of *C. plica* in Denmark. The *C. plica* prevalence of Danish red foxes correspond with a study from Germany with a prevalence of 78% in red foxes (Bork-Mimm and Rinder, 2011). However, the prevalence in our study was lower than in Lithuania (98,3%) and higher than in Norway (53.0%) and Hungary (52.0%) (Bork-Mimm and Rinder, 2011; Bružinskaitė-Schmidhalter et al., 2012; Davidson et al., 2006; Magi et al., 2015; Saeed et al., 2006). In addition, red foxes are becoming increasingly more “urbanized” wildlife (Deplazes et al., 2004; Gloor et al., 2002; Scott et al., 2014), carrying the parasite close to premises where pet animals are roaming. Thus, red foxes are seemingly the most important factor in the epidemiology and risk of spread of the parasite to pet dogs.

In our study, the *C. plica* prevalence in raccoon dogs (0.5%) is considerably lower than in red foxes (73.7%). This observation correspond to a Lithuanian study documenting a prevalence of 11.3% in raccoon dogs and 93.3% in red foxes (Bružinskaitė-Schmidhalter et al., 2012). To our knowledge, the Lithuanian study is the only other study where *C. plica* infection in raccoon dogs have been analysed.

Ingestion of infected earthworms is the sole documented infection method for *C. plica* in canines. Elmeros et al. (2018) and Mikkelsen et al. (2016) demonstrated that Danish raccoon dogs to a large extent eat earthworms (68.6% and 34.7% of animals). In contrast, red foxes rarely eat earthworms, but prefer microtine rodents (Kauhala et al., 1998; Pagh et al., 2015a, 2015b). Hence, the *C. plica* prevalence herein observed for both raccoon dogs and red foxes contradicts the feed preference. This could indicate that raccoon dogs are less suitable as final host for *C. plica.* This applies for badgers, too. A Spanish study documented a prevalence of 2.1% in Eurasian badgers (Torres et al., 2001), although badgers frequently consume earthworms (Kauhala et al., 1998; Madsen et al., 2002). In addition to acquire the infection through consumption of earthworms, it is likely that red foxes can acquire the infection through paratenic hosts like rodents and birds. When rodents and birds consume *C. plica*-infected earthworms, the parasite seemingly enters these animals without undergoing any further development, but might remain alive till it gains entry to the red fox through consumption of the paratenic host. However, further studies are needed to identify paratenic hosts. A second possibility is that another, hitherto undocumented intermediate host exist. Lastly, *C. plica* infection might accumulate in red fox, so once infected, the parasite reside within the bladder for an extended time. This could be the case if an age effect was noted. Unfortunately, the age was not recorded in this study. A last possibility is that *Capillaria* eggs documented from the various hosts are indeed different species. However, our study was limited by lack of molecular analysis of *Capillaria* eggs from the various species.

The red foxes from Jutland had significantly higher *C. plica* prevalence compared to red foxes form Zealand. Jutland is the peninsula that forms the continental portion of Denmark. The population density is 84 persons per km^2^. In comparison, the population density is 358 persons per km^2^ on Zealand. Since only few red foxes originated from Zealand, we refrain to conclude that the difference in prevalence between regions is affected by the availability of infected earthworms or the feeding habits of red foxes.

This is the first report of *C. plica* infection in beech martens (4 positive out of 20). *Capillaria plica* have been recorded in American martens with a prevalence of 6% (Seville and Addison, 1995), which determine that the genus *martes* are final hosts of *C. plica*.

In summary, our findings confirm that most Danish red foxes excrete *C. plica* eggs and that red foxes are the most likely reservoir host for infection in dogs. Moreover, further studies are needed to identify other possible intermediate hosts and infection through transport hosts.

## Appendix A Supplementary data

Data Profile Data Profile
